# Atmospheric Oxygen Tension Slows Myoblast Proliferation via Mitochondrial Activation

**DOI:** 10.1371/journal.pone.0043853

**Published:** 2012-08-24

**Authors:** Stephanie Duguez, William J. Duddy, Viola Gnocchi, James Bowe, Sherry Dadgar, Terence A. Partridge

**Affiliations:** 1 Research Center for Genetic Medicine, Children's National Medical Center, Washington, District of Columbia, United States of America; 2 Université Pierre et Marie Curie (UPMC UMR S 974)-Institut National de la Santé et de la Recherche Médicale (Inserm U974)-Centre National de la Recherche Scientifique (CNRS UMR 7215), Institut de Myologie, Paris, France; University of Frankfurt - University Hospital Frankfurt, Germany

## Abstract

**Background:**

Mitochondrial activity inhibits proliferation and is required for differentiation of myoblasts. Myoblast proliferation is also inhibited by the ∼20% oxygen level used in standard tissue culture. We hypothesize that mitochondrial activity would be greater at hyperoxia (20% O_2_) relative to more physiological oxygen (5% O_2_).

**Methodology/Principal Findings:**

Murine primary myoblasts from isolated myofibres and conditionally immortalized H-2K myoblasts were cultured at 5% and 20% oxygen. Proliferation, assayed by cell counts, EdU labeling, and CFSE dilution, was slower at 20% oxygen. Expression of MyoD in primary myoblasts was delayed at 20% oxygen, but myogenicity, as measured by fusion index, was slightly higher. FACS-based measurement of mitochondrial activity indicators and luminometric measurement of ATP levels revealed that mitochondria exhibited greater membrane potential and higher levels of Reactive Oxygen Species (ROS) at 20% oxygen with concomitant elevation of intracellular ATP. Mitochondrial mass was unaffected. Low concentrations of CCCP, a respiratory chain uncoupler, and Oligomycin A, an ATP synthase inhibitor, each increased the rate of myoblast proliferation. ROS were investigated as a potential mechanism of mitochondrial retrograde signaling, but scavenging of ROS levels by N-acetyl-cysteine (NAC) or α-Phenyl-N-tert-butylnitrone (PBN) did not rescue the suppressed rate of cell division in hyperoxic conditions, suggesting other pathways. Primary myoblasts from older mice showed a slower proliferation than those from younger adult mice at 20% oxygen but no difference at 5% oxygen.

**Conclusions/Significance:**

These results implicate mitochondrial regulation as a mechanistic explanation for myoblast response to oxygen tension. The rescue of proliferation rate in myoblasts of aged mice by 5% oxygen suggests a major artefactual component to age-related decline of satellite cell proliferation in standard tissue culture at 20% oxygen. It lends weight to the idea that these age-related changes result at least in part from environmental factors rather than characteristics intrinsic to the satellite cell.

## Introduction

Proliferation of myoblasts, both murine and human, is inhibited by ∼20% oxygen tensions [1], [2], [3], [4]. Despite this, the gaseous environment of myoblast tissue culture is standardly uncontrolled except for carbon dioxide buffering. Normal physiological oxygen levels in muscle tissue are estimated to be 10.0 to 28.8 mmHg [5], whereas, at sea level, standard tissue culture gas consisting of 95% ambient air plus 5% CO_2_ contains ∼142 mmHg of oxygen [3]. Inhibition of myoblast proliferation at ∼20% oxygen tensions (to which we refer as ‘hyperoxia’) compared with more physiological oxygen (6%) was first noted in cultures of murine primary myoblasts grown from single myofibres [1]. Inhibition of proliferation was associated with a reduced expression of myogenic transcription factors Myf5, MyoD, and myogenin and a small proportion of cells showed greater lipid accumulation, leading the authors to propose a switch from myogenic to adipogenic cell fate. Manipulation of levels of oxygen and of reactive oxygen species (ROS) during culture of the spontaneously immortal C2C12 murine myoblast line show reduced expression of myogenic transcription factors and slower differentiation under oxidative conditions, with the converse under reducing conditions [2]. However, a study using human skeletal muscle precursor cells, despite showing an inhibition of proliferation under hyperoxia, observed little effect on MyoD and myogenin expression and detected no evidence of adipogenesis [4]. Consistent with this latter report, a recent analysis of myoblast cultures from isolated myofibres of the *MyoD^iCre^* knockin mouse, in which satellite cells are permanently labeled following developmental expression of MyoD, demonstrated retention of myogenicity with no spontaneous adipogenic differentiation under hyperoxic conditions [6]. In the absence of a switch to adipogenesis, mechanisms underlying the myoblast response to hyperoxia remain to be elucidated.

Study of cell physiology during proliferation and differentiation has seen the emergence of mitochondria as important regulators. More recent work has built upon the early observation by Warburg [7] that the proliferation of cancer cells is associated with a switch from mitochondrial ATP production via oxidative phosphorylation to glycolytic ATP production. Several mechanisms have been described that mediate retrograde signaling from mitochondria to the cell nucleus [8], [9], [10] and, in myoblasts, mitochondrial activity has been associated with regulation of proliferation [11], [12], [13] and differentiation [13], [14], [15], [16], [17], [18]. Cell cycle analyses of the myoblast response to increased mitochondrial activity [19] and to hyperoxia [3] suggest a common mechanistic link as the response in both cases involves upregulation of the cyclin-dependent kinase inhibitor, p21 [3], [4], [19].

We hypothesized that greater mitochondrial activity is associated with myoblast responses to hyperoxia. We used two myoblast cell culture models: primary myoblasts cultured from matrigel-adhered freshly isolated myofibres [20], and conditionally immortalized H-2K myoblasts [21]. Initially we showed that the inhibition of proliferation by hyperoxia occurs during culture of cells from both adult (16–20 weeks old) and aged (32–40 weeks old) mice. We then tested the hypothesis that myogenicity is lost under hyperoxic conditions, as proposed previously [1], by assaying the fusion of myoblasts to newly formed myotubes. Rather than a loss of myogenic fusion, we observed an increase, consistent with the involvement of mitochondrial activity in cell cycle exit [13], [14], [15] and leading us to explore mitochondrial activity, ROS levels, ATP levels, and finally to test whether modifiers of ROS levels, H_2_O_2_, N-acetyl-cysteine (NAC), and α-Phenyl-N-tert-butylnitrone (PBN), can exacerbate or rescue the inhibitory effects of hyperoxia.

## Materials and Methods

### Animal use and ethics

Two mouse strains were used in this study: wild-type C57BL/10SnJ, obtained from JAX mice, and the transgenic strain H-2K^b^-tsA58 (CBA/ca X C57Bl10), obtained from Charles River, were used at the ages indicated in the Results section. All animal procedures were performed according to Children's National Medical Center Institutional Animal Care and Use Committee (IACUC) and National Institutes of Health guidelines, and were specifically approved under protocol number 242-09-06 of Children's National Medical Center IACUC. All efforts were made to minimize suffering.

### Myofibre isolation

Reagents were from Invitrogen unless otherwise stated. Single myofibres were isolated as described previously [20]. Briefly: extensor digitorum longus (EDL) muscles were carefully dissected immediately after euthanizing the mouse; connective tissue was digested by incubating each muscle in 0.2% Collagenase Type 1 (Sigma) in DMEM for 1 to 2 hours depending on muscle size; single myofibres were liberated by trituration with fire-smoothed wide-mouthed Pasteur pipettes in dishes containing DMEM and pre-coated with horse serum (to prevent adherence); liberated myofibres were washed by serial transfer through four such dishes.

### Primary myoblast culture, oxygen regulation, and EdU labeling

Pure cultures of primary myoblasts were obtained by tissue culture of isolated single myofibres as described previously [20]. Briefly: a single drop of 1 mg/ml Matrigel (BD Biosciences) was used to coat each well of a 24-well plate (BD Biosciences); after 30 minutes at 37°C, excess matrigel was removed and one myofibre was placed in each well followed by 0.5 ml of plating medium comprised of DMEM (with 4,500 mg/L D-glucose, L-glutamine, and 110 mg/L sodium pyruvate) with 10% v/v horse serum, 2% v/v (4 mM) L-glutamine (Sigma), and 1% v/v Penicillin/Streptomycin (Sigma). Cells were then maintained in an atmosphere controlled at 20% or 5% oxygen, and 5% CO_2_. For the analysis of primary myoblast proliferation, 10 µM 5-ethynyl-2′-deoxyuridine (EdU) was added to culture medium. For oxygen scavenging experiments, n-acetyl-cysteine (NAC; Sigma) was added at concentrations specified. At time-points indicated, cells were fixed by addition of 100 µl of 100% formaldehyde to the culture medium, so as not to disturb the myofibre which is often only loosely attached prior to fixation, then stored at 4°C for one to several days prior to staining. EdU-labeled cells were stained using Click-iT® EdU Alexa Fluor 594 Imaging Kit according to the manufacturer's instructions. Briefly: after fixation, cells were permeabilized with 0.5% Triton in PBS for 20 minutes at room temperature, then incubated with the EdU Click-iT reaction cocktail for 30 minutes at room temperature. Following 3 rinses with TBS-tween, DAPI was applied for 15 minutes at 0.5 µg/ml. Visualization and imaging were carried out using a Nikon Eclipse E500 epifluorescence microscope.

### Fusion Index

At day 3 of primary myoblast proliferation, the myofibre was physically detached using a pipette tip and the plating medium was removed and replaced with 0.5 ml proliferation medium (DMEM with 20% v/v foetal bovine serum, 2% v/v chick embryo extract (Accurate), 2% v/v (4 mM) L-glutamine (Sigma), and 1% v/v Penicillin/Streptomycin (Sigma)). After a further 3 days of culture, proliferation medium was replaced with differentiation medium (DMEM with 5% v/v foetal bovine serum, 2% v/v (4 mM) L-glutamine (Sigma), and 1% v/v Penicillin/Streptomycin (Sigma)). Following 5 days of differentiation, cells were fixed and stained with DAPI. Fusion index was then calculated as the percentage of nuclei contained within myotubes (defined as a cell containing 3 or more nuclei). Means and confidence intervals are given for five wells at each oxygen concentration.

### Immunohistochemical staining of primary myoblasts on their resident myofibre

For assay of Pax7 and MyoD activation, myoblasts were stained on their resident myofibre. Myofibres were either fixed in 3.65% Formaldehyde at 37°C for 15 minutes immediately following isolation or maintained in suspension in plating medium then fixed. Myofibres were then stored in PBS at 4°C for one or several days prior to staining. Permeabilization and blocking buffer (TBS-tween with Goat serum 20%, BSA 2%, Triton 0.5%, and Tween 0.1%) was applied for 30 minutes at room temperature then cells were left in primary antibody solution (mouse IgG1 anti-MyoD (Vector labs; 1∶50 dilution in PBS with BSA 2%) or mouse IgG1 anti-Pax7 (Developmental Studies Hybridoma Bank; undiluted)) overnight at 4°C. Cells were rinsed 3 times in TBS-tween (0.1%), then submerged in secondary antibody solution (Alexa Fluor-488 anti-mouse IgG1 (1∶400 dilution)) for 1 hour at room temperature. Following a further 3 rinses, DAPI was applied for 15 minutes at 0.5 µg/ml. Visualization and imaging were carried out using a Nikon Eclipse E500 epifluorescence microscope.

### Derivation of H-2K myoblasts

We have used a conditionally immortalized H-2K myoblast cell line to facilitate experiments that require large numbers of cells. Immortalized H-2K myoblasts were obtained as described previously [21] with minor modifications. Briefly, extensor digitorum longus (EDL) muscles were removed from male H-2K^b^-tsA58 (CBA/ca X C57Bl6) mice at 1 month of age. Single myofibres were obtained and adhered to Matrigel in 24-well plates, as described above. Myofibres were cultured at 33°C 10% CO_2_, in proliferation medium with γ-interferon (2 units/ml). Under these conditions, immortality is maintained as γ-interferon drives expression of a thermolabile simian virus 40 (SV40) large tumor (T) antigen, which blocks exit from the cell cycle [22]. When a single myoblast had migrated from the myofibre, the myofibre was removed. The clone was then expanded and myogenicity was confirmed by culture at 37°C 5% CO_2_ in differentiation medium, checking for myotube formation and, at 8 days, Dystrophin expression. Due to this clonal derivation method, there is no selection of cellular sub-populations in the proliferating pool, a possible disadvantage that cannot be discounted when growing up large numbers of non-immortalized primary myoblasts from isolated myofibres or whole muscle dissociations. Myoblasts generated by use of this protocol form normal muscle when grafted into recipient mdx mice, even after extensive *in vitro* expansion [21]. To further diminish the chances of accumulating anomalies such as aberrant caryotypes as occurs with longer established spontaneously immortalized lines such as the C2C12, we used low passage numbers (<14) throughout.

### Culture of H-2K myoblasts and CFSE analysis using FACS

H-2K myoblasts were cultured under immortalizing conditions (33°C in the presence of γ-interferon) then trypsinized and stained with 10 µM carboxyfluorescein diacetate, succinimidyl ester (CFSE) according to the manufacturer's instructions. They were then cultured at 37°C without γ-interferon for 3 days at 5% oxygen, or 20% oxygen, with or without a treatment compound (30 nM NAC, 1.5–5 nM carbonyl cyanide m-chlorophenyl hydrazone (CCCP; from Sigma), 0.0005–0.05 µg/ml Oligomycin A (Sigma), 0.5–100 µM H_2_O_2_ (Sigma), or 10 nM to 1 µM α-Phenyl-N-tert-butylnitrone (PBN; Sigma)). Cells were then trypsinized and fixed in 3.65% formaldehyde prior to analysis by Fluorescence-activated cell sorting (FACS). CFSE data were deconvoluted and proliferation index was calculated using FlowJo software version 9.4.10. Almost all cells divided at least once (99.4% and 94.6% at 5% and 20% oxygen, respectively). For three different experiments comparing (1) 5% with 20% oxygen, (2) untreated at 20% oxygen with 1.5 nmol CCCP treated at 20% oxygen, and (3) untreated at 20% oxygen with 0.005 µg/ml Oligomycin A treated at 20% oxygen, values for each parameter used for deconvolution of CFSE data are now given, respectively: mean signal intensity of the undivided population was (1) 3,338, (2) 2,890, and (3) 4,840; the ratio of signal between successive peaks was fixed at (1) 0.504, (2) 0.501, and (3) 0.495; the cv of each peak was fixed at (1) 6.72, (2) 5.99, and (3) 6.00. With those parameters, Root Mean Squares were (1) 1.68, (2) 2.90, and (3) 3.54.

### Analysis of H-2K cell mitochondrial membrane potential, mitochondrial mass and H_2_O_2_ levels using FACS

For the analysis of mitochondrial membrane potential and mitochondrial mass, H-2K myoblasts were cultured for 3 days at 37°C, under 5% or 20% oxygen levels, without γ-interferon. Cells were trypsinized, washed and resuspended in DMEM, and incubated for 30 minutes at 37°C with either: 3,3′-dihexyloxacarbocyanine iodide (DioC_6_; from Sigma) at 100 nM, nonyl-acridine orange (NAO) at 10 µM, and were then subjected to FACS.

To measure intracellular levels of H_2_O_2_, H-2K myoblasts were cultured as described above with or without the addition of CCCP, Oligomycin A, PBN, or H_2_O_2_. Cells were harvested after incubation for 30 minutes at 37°C with dichlorodihydrofluorescein diacetate (H_2_DCFDA; from Sigma) at 50 µM. To determine whether H_2_O_2_ produced by cells cultured at 20% oxygen came from the mitochondria, cells were incubated for 30 min at 37°C with H_2_DCFDA at 50 µM and CCCP at 4 mM, and were subjected to FACS.

For all FACS analyses, labeling with 7-AAD was used to detect dead cells which were then excluded from the analysis. Side- and forward-scatter were used to select cell-sized objects.

For optimization of oxygen scavenging assay, NAC was added at the commencement of experimental culture conditions, at concentrations indicated, and H_2_O_2_ level was measured with H_2_DCFDA at day 3.

### Assay of intracellular ATP level

H-2K myoblasts were cultured to sufficient numbers under immortalizing conditions then transferred to experimental conditions for 3 days. Cells were trypsinized and centrifuged at 1000 g for 3 min at 4°C. Cell pellets were washed three times by resuspension in cold PBS followed by centrifugation for 3 min at 4°C. Pellets were resuspended in 16.3 µl of cold 70% perchloric acid and 80 µl of cold PBS was added. The suspensions were vortexed, incubated for 10 min at 4°C, and centrifuged at 10,000 g for 20 min at 4°C to pellet non-soluble material. The supernatant containing ATP was transferred to a fresh vial and 110.4 µl of cold 2 M KOH was added. Samples were then centrifuged at 10,000 g for 20 min at 4°C and stored at −80°C. The ATP bioluminescence was quantified using a Mithras LB 940 bioluminometer (Berthold Technologies) and the ATP determination kit (Molecular Probe, Invitrogen) according to the manufacturer's instructions. Briefly, after measuring the bioluminescence background from 180 µl of assay buffer, 20 µl of sample was added and the bioluminescence produced by the luciferase was read for 50 seconds.

### Statistical Analyses

Student's T-test was used throughout unless otherwise stated. Figure captions indicate p-values.

## Results

### Effect of hyperoxia on cell counts of primary myoblasts from isolated myofibres

**Figure 1:**
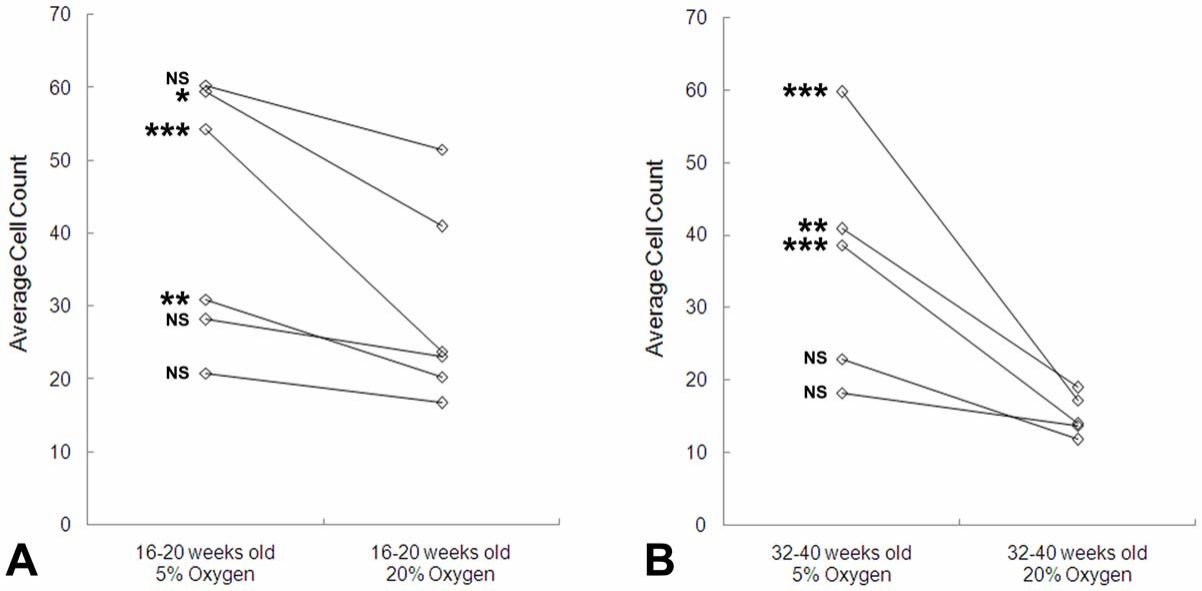
Hyperoxia reduces cell counts following 3 days of primary myoblast culture. Pure myoblast populations were cultured from single myofibres isolated from (A) adult 16–20 week old and (B) aged 32–40 week old mice. Each pair of data points represents average cell counts from myofibres of one EDL muscle of a single animal. Cells from an average of 20 myofibres were counted per data point (i.e. average n≈20). Student's t-test: NS = not significant; * = P<0.05; ** = P<0.01; *** = P<0.001.

It was previously reported that counts of primary myoblasts are reduced under hyperoxic conditions (20% oxygen) compared with more physiological (6%) oxygen following 3 days of culture from single myofibres isolated from the EDL muscles of 14–28 week old mice [1]. To confirm this and to investigate the effect of aging, myofibres were isolated from adult (16–20 weeks old) and aged (32–40 weeks old) mice. Myofibres were adhered to a Matrigel substrate which facilitates myoblast migration and proliferation. Each myofibre was placed into a single well of a 24-well plate, and the number of myoblasts was counted following 3 days of tissue culture at either 5% or 20% oxygen. Data are presented in which each pair of data points represents average values from a single experiment involving all of the myofibres obtained from one animal (Fig. 1). At both ages considerable mouse to mouse variation was observed, leading us to carry out a total of 11 experiments with cell counts from more than 400 myofibres. In each of these experiments, average cell counts were lower at 20% than 5% oxygen, with varying degrees of statistical significance. The more pronounced effect was observed with myoblasts from aged mice. Averaging across experiments, the cell counts were 29% (SD 15.5) lower at 20% than at 5% among satellite cells from adult mice, and 52% (SD 17.7) lower among those from aged mice. Interestingly, the range of average values observed at 5% oxygen was similar at the two ages, the major difference between ages being that the aged group showed consistently and significantly lower counts at 20% oxygen.

### Myogenicity is not lost under hyperoxia

**Figure 2:**
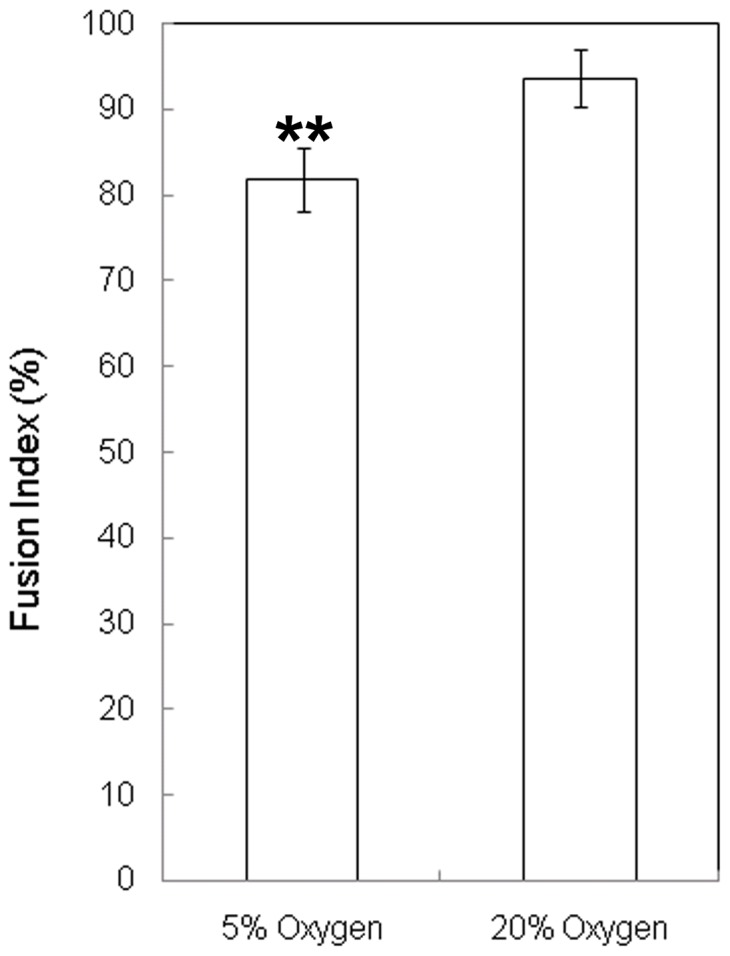
Myogenicity is slightly increased under hyperoxia. Fusion Index is calculated as the percentage of nuclei contributing to multi-nucleated cells after 5 days of differentiation. Confidence intervals (95%) are shown. Student's t-test: ** = P<0.01 (n = 5).

It was previously suggested that the reduced cell counts of myoblasts cultured under hyperoxia result from loss of myogenicity associated with increased adiposity [1]. To test myogenicity, we measured the fusion index of primary myoblasts proliferated and differentiated at 5% or 20% oxygen. Myoblasts from single myofibres of an adult mouse were grown for six days under proliferation conditions, then switched to differentiation conditions for five days, and the number of nuclei contributing to myotubes were counted. The original myofibre was physically detached at day 3 of proliferation to prevent its fusion with newly formed myotubes. A small but significant increase in fusion was observed at 20% compared with 5% oxygen (Fig. 2). We did not detect any Oil Red O staining or morphological suggestion of lipid vesicles at 5% or 20% oxygen in myoblasts or myotubes (data not shown).

### H-2K myoblast proliferation is inhibited by hyperoxia

**Figure 3:**
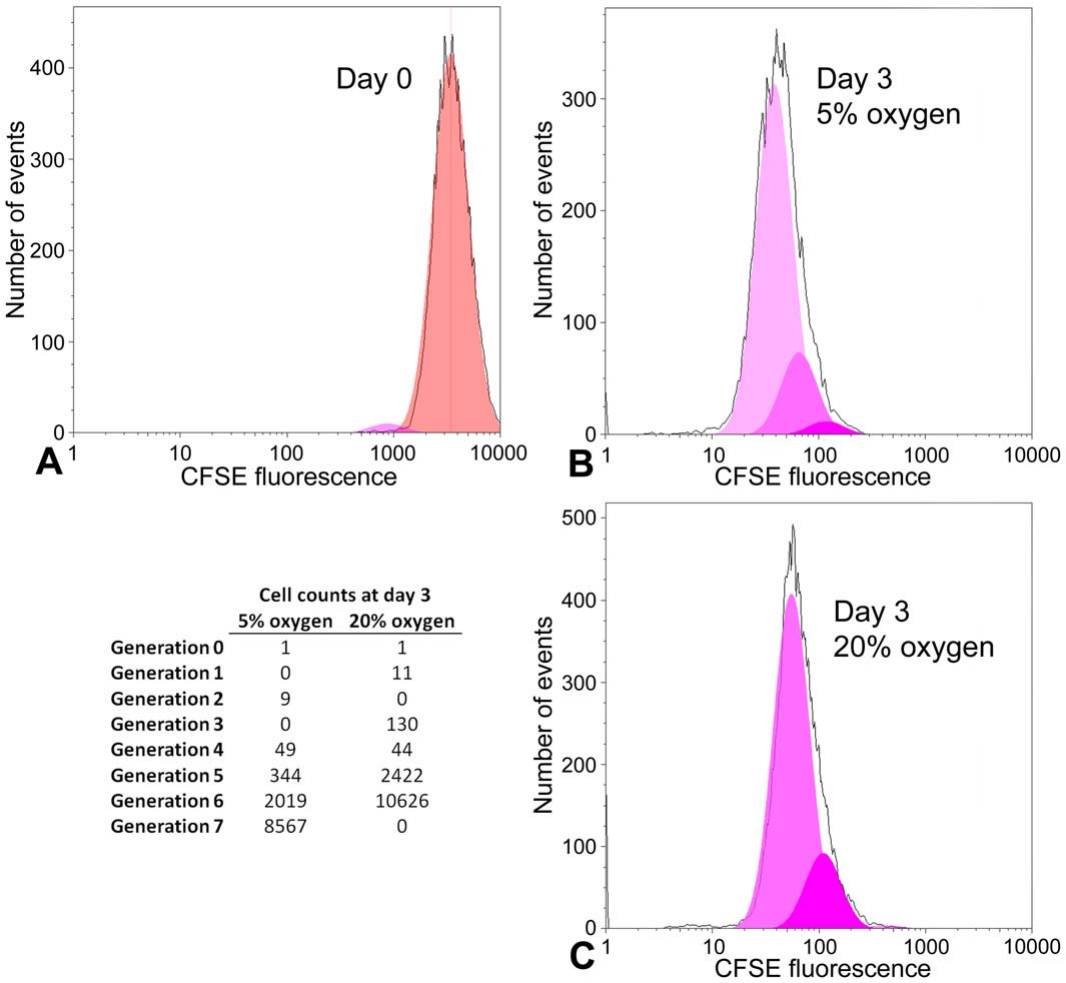
Inhibition of H-2K myoblast proliferation by hyperoxia. Cells were stained with CFSE and cultured for 3 days at 5% or 20% oxygen. The number of cells of each generation was estimated by deconvolution of FACS data. Total cell counts (plotted in black) and modeled generational subsets (coloured curves) are shown immediately following CFSE staining (A), and following 3 days of culture at 5% oxygen (B) and 20% oxygen (C). Cell counts are also shown in tabulated form. Approximately ten thousand cells were analyzed per condition. After 3 days cells cultured at 20% oxygen had undergone an average of approximately one less division compared with 5% oxygen.

We clonally isolated conditionally immortal myoblasts from the H-2K^b^-tsA58 mouse, to obtain large numbers of a pure myoblast population, as described under materials and methods. To measure H-2K cell proliferation rate we used CFSE to fluorescently label cytoplasmic proteins. Cells were uniformly labeled with CFSE, which then equally distributes between daughter cells with each round of cell division, facilitating estimation of proliferation rate by mathematical deconvolution of FACS data. H-2K cells showed a more rapid dilution of CFSE signal when cultured at 5% oxygen (Fig. 3), with a proliferation index estimated at 6.50 after 3 days, compared with 5.45 at 20% oxygen.

### Hyperoxia induces increased mitochondrial respiratory activity of H-2K myoblasts: assay of mitochondrial proton gradient, mitochondrial mass, ROS, and ATP levels

**Figure 4:**
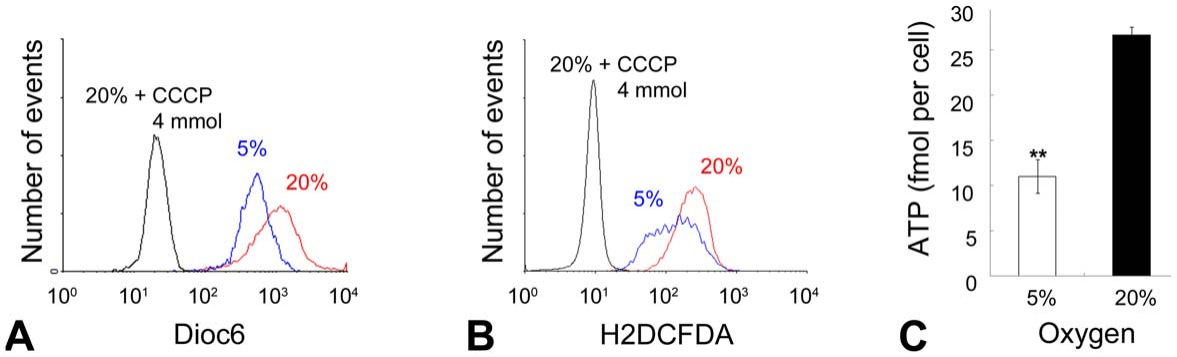
Mitochondrial activity, H_2_O_2_ level, and intracellular ATP are increased under hyperoxia. Representative plots show, (A) Dioc6 fluorescence indicative of proton gradient and, (B) H_2_DCFDA fluorescence indicative of ROS levels, of cells cultured at 5% and 20% oxygen. Signals from Dioc6 and H_2_DCFDA were both confirmed as mitochondrial due to their abrogation by the respiratory chain decoupling agent, CCCP. (C) Intracellular ATP is increased under hyperoxia. Cells were cultured for 3 days then ATP level was measured using a luciferase luminance assay. Confidence intervals (95%) are shown. Student's t-test: ** = P<0.01 (n = 3).

We speculated that oxygen levels might directly influence mitochondrial metabolism and that this could provide a mechanistic explanation for the observed differences in myogenic commitment and rate of proliferation. To investigate this we used FACS analysis to measure cellular uptake of the small fluorescent molecule, Dioc6, accumulation of which is dependent upon proton gradient (ΔΨ)across the mitochondrial membrane [23]. To obtain sufficient cells for FACS analysis we used H-2K myoblasts. Average Dioc6 signal following 3 days of proliferation was 1.6-fold greater under hyperoxia than under physiological oxygen (Fig. 4A). This proton gradient was entirely ablated by the respiratory chain uncoupler, CCCP, indicating that the increased ΔΨ is of mitochondrial origin. Mitochondrial mass, as detected by NAO fluorescence (data not shown), was unchanged.

One way in which an increased proton gradient may act to alter cell behaviour such as activation and proliferation is via increased synthesis of Reactive Oxygen Species (ROS). To test whether levels of ROS are greater under hyperoxia we used FACS analysis to measure fluorescently activated H_2_DCFDA, an indicator of H_2_O_2_ levels, finding 2.6-fold higher signal under hyperoxia than under physiological oxygen, raising the possibility of ROS synthesis as a mechanism by which myoblast activation may be inhibited (Fig. 4B). Since ROS may be synthesized outside the mitochondria we used CCCP to uncouple the respiratory chain under hyperoxia, resulting in complete ablation of H_2_DCFDA fluorescence, indicating that the observed H_2_O_2_ level is dependent upon ΔΨ [24].

Another potential consequence of altered mitochondrial activity and a possible mechanistic link with cell proliferation rate is the intracellular level of ATP. Our luminometric assay of ATP levels with luciferase as a reporter, showed, after 3 days culture that intracellular ATP level at 20% oxygen was more than double that at 5% (Fig. 4C).

### Uncoupling of the electron transport chain or inhibition of ATP synthase increases the rate of H-2K myoblast proliferation

**Figure 5:**
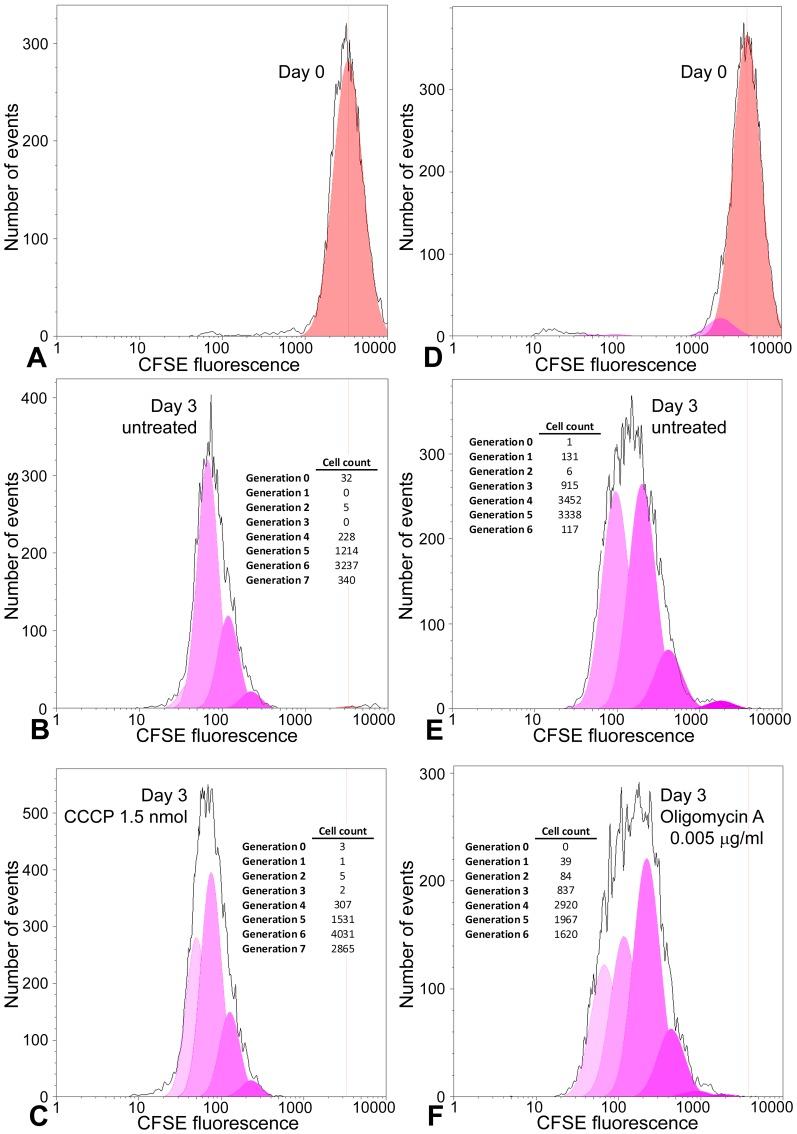
Uncoupling of the respiratory chain or inhibition of ATP synthase increases the rate of H-2K myoblast proliferation. Cells were stained with CFSE and cultured for 3 days with low concentrations of CCCP or Oligomycin A, or respective vehicle controls. The number of cells of each generation was estimated by deconvolution of FACS data. Total cell counts (plotted in black) and modeled generational subsets (coloured curves) are shown at day 0 immediately following CFSE staining (A and D represent day 0 for CCCP and Oligomycin A experiments, respectively), and following 3 days of culture with vehicle only (B and E for CCCP and Oligomycon A experiments, respectively) or with treatment (C and F for CCCP 1.5 nmol and Oligomycin A 0.005 µg/ml, respectively). Cell counts are also shown in tabulated form. Approximately ten thousand cells were analyzed per condition.

To test for a mechanistic link between mitochondrial activity and myoblast proliferation rate, H-2K myoblasts were treated with a range of concentrations of CCCP, an uncoupler of the respiratory chain, or of Oligomycin A, an inhibitor of ATP synthase. Following 3 days of culture at 20% oxygen, the rate of proliferation was increased at low concentrations of CCCP (1.5–5 nmol) and of Oligomycin A (0.0005–0.05 µg/ml). Deconvoluted CFSE data are shown for CCCP at 1.5 nmol (Fig. 5A–C) and Oligomycin A at 0.005 µg/ml (Fig. 5D–F). Levels of intracellular H_2_O_2_ and ATP were measured across these concentration ranges (table 1). H_2_DCFDA fluorescence, indicating H_2_O_2_ level, was reduced by treatment with either CCCP or Oligomycin A, with decreases ranging from 28–68% and 66–94%, respectively. Statistical significance was high at all concentrations, except for 5 nmol CCCP, at which the difference was not significant. Levels of intracellular ATP were lower at all concentrations of CCCP and Oligomycin A that were tested, though differences were in some cases small and not always statistically significant (table 1). Oligomycin A treatment at 5% oxygen had no effect on proliferation rate (Fig. S1).

**Table 1:** Intracellular H_2_O_2_ (H_2_DCFDA fluorescence signal) and ATP levels of H-2K myoblasts in response to treatment with low concentrations of CCCP and Oligomycin A.

Condition	Mean H_2_DCFDA fluorescence±sd	Mean intracellular ATP (fmol/cell)±sd
Untreated	541.67±36.07	23.97±0.17
CCCP 1.5 nmol	233.33±35.53***	19.15±2.56^ns^
CCCP 2 nmol	178.33±35.00***	13.97±1.36*
CCCP 5 nmol	385.00±99.23^ns^	20.35±0.51*
Oligomycin A 0.0005 ìg/ml	181.33±53.82**	21.08±0.51^ns^
Oligomycin A 0.005 ìg/ml	38.93±1.70**	12.16±1.19*
Oligomycin A 0.05 ìg/ml	36.03±2.54**	14.21±1.36*

Student's t-test v untreated: * = P<0.05; ** = P<0.01; *** = P<0.001; ns = not significant

### Inhibition of H-2K proliferation by hyperoxia is not ameliorated by ROS scavenging

**Figure 6:**
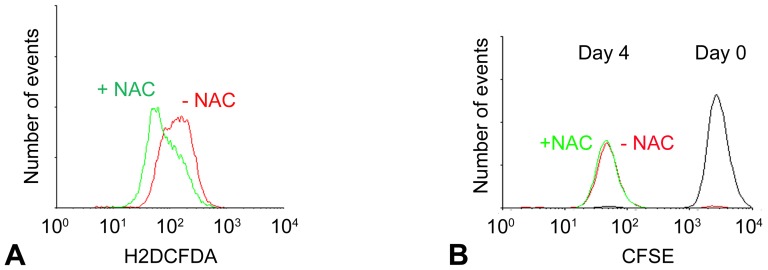
ROS scavenging by n-acetyl-cysteine does not ameliorate proliferation rate of H-2K myoblasts under hyperoxia. Representative plots show that (A) ROS levels of live H-2K cells cultured at 20% oxygen were rescued to levels similar to 5% oxygen by administration of NAC at 30 nM and (B) assay of proliferation rate using CFSE showed no effect of NAC after 4 days of culture.

**Figure 7:**
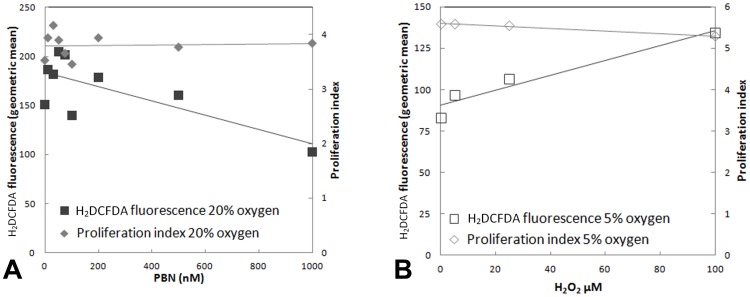
Manipulation of ROS levels using PBN at 20% oxygen or H_2_O_2_ at 5% oxygen does not change the proliferation rate of H-2K myoblasts. ROS levels, as indicated by the geometric mean of H_2_DCFDA fluorescence determined by FACS analysis (primary y-axes), and proliferation indices, as measured using CFSE (secondary y-axes) are plotted against concentrations of (A) PBN or (B) H_2_O_2_, for H-2K myoblasts cultured for 3 days at 20% or 5% oxygen, respectively. Lines of best fit are shown.

If increased ROS were involved in the inhibition of proliferation under hyperoxia then scavenging of ROS using n-acetyl-cysteine (NAC) or PBN may be expected to relax this inhibition. H-2K cells cultured under hyperoxia were administered NAC at a range of concentrations (20, 30, 40, 100, and 1000 nM) and ROS levels were measured by FACS analysis of H_2_DCFDA fluorescence. Maximal scavenging of ROS was observed at 30 nM NAC (data not shown), administration at this concentration reduced ROS levels close to those at 5% oxygen (Fig. 6A), but administration of this same concentration throughout a CFSE-based assay of proliferation, produced no effect (Fig. 6B). Similarly, no effect was observed on proliferation index in a CFSE proliferation assay using PBN at nine different concentrations ranging from 10 nM to 1 µM, despite a decrease in levels of H_2_DCFDA fluorescence (Fig. 7A; deconvolutions of CFSE data are shown in Fig. S2).

We tested for suppression of proliferation rate at 5% oxygen by H_2_O_2_ at concentrations of 5, 25, and 100 µM, Levels of H_2_DCFDA fluorescence were increased but proliferation index was unchanged (Fig. 7B; deconvoluted CFSE data are shown Fig. S3). At 20% oxygen, H_2_DCFDA fluorescence correlated with increasing H_2_O_2_ levels and minimal effects were observed on proliferation index (Fig. S4 and Fig. S5), except at the two higher concentrations, 25 and 100 µM, which induced a marked slowing of proliferation, but this was associated with abundant cell death (which was not observed in the other H_2_O_2_ conditions) so this may reflect the effects of sub-lethal toxicity on surviving cells under the stress of hyperoxia rather than the effects of ROS-mediated signaling.

### Effects of oxygen and ROS scavenging on primary myoblast activation and proliferation

**Figure 8:**
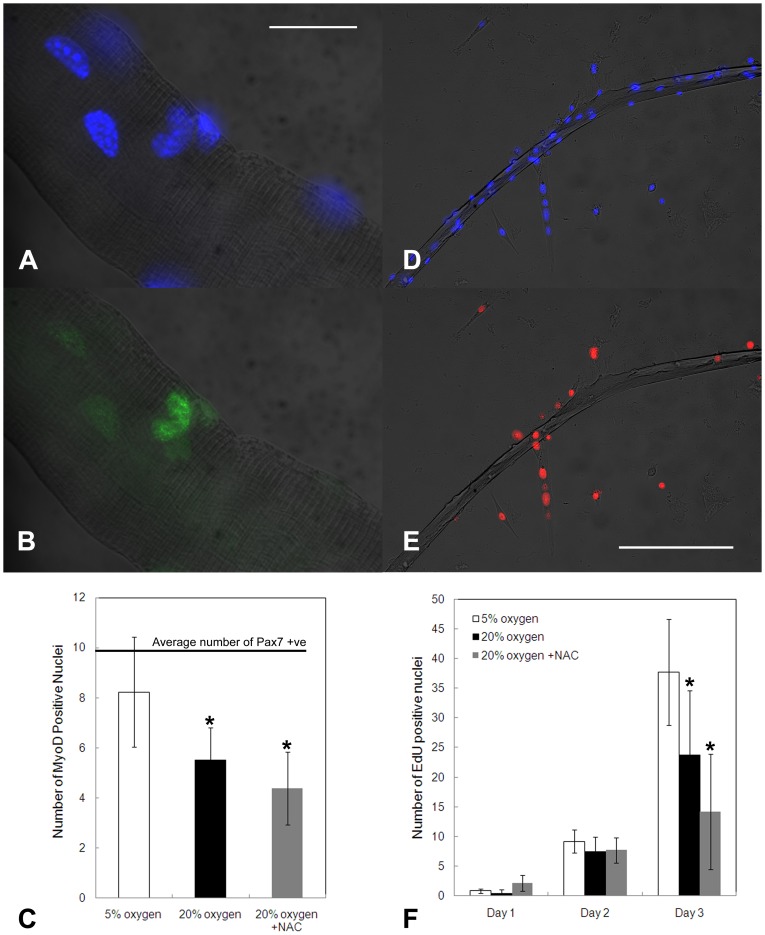
Hyperoxia inhibits MyoD expression during early activation and inhibits early rounds of proliferation. (A, B, C) Effect on early activation. (A, B) Transmitted light micrograph of isolated myofibre overlaid with nuclear (DAPI; blue) and MyoD (green) staining, respectively. Scale bar = 20 µm. (C) Average number of activated myoblasts (MyoD positive nuclei) per myofibre 4 hours after isolation and culture was fewer at 20% than 5% oxygen. Also indicated is the average number of quiescent myoblasts (Pax7 positive nuclei) immediately following isolation of a separate batch of myofibres from the same muscle. (D, E, F) Effect on early rounds of proliferation. (D, E) Transmitted light micrograph of an isolated matrigel-adhered myofibre and associated myoblasts overlaid with nuclear stain (DAPI; blue) and proliferated cell stain (EdU positive nuclei; red), respectively. Scale bar = 200 µm. (F) The thymidine analogue EdUwas present in medium throughout cell culture, thereby marking all daughter cells. Delay of proliferation by hyperoxia emerges between days 2 and 3 of cell culture. Oxygen scavenging by NAC had no significant effect on activation or early proliferation compared with hyperoxia alone. Confidence intervals (95%) are shown. Student's t-test: * = P<0.05 (compared with value for 5% oxygen).

To determine whether hyperoxia inhibits myoblast activation we counted the number per myofibre of nuclei expressing MyoD, an early marker of myogenic commitment, at 4 hours after myofibre isolation. The average number of myoblasts present per myofibre was determined by immunostaining against the quiescent myoblast marker, Pax7, immediately after myofibre isolation. At 4 hours, more than 83% of myoblasts expressed MyoD under physiological oxygen, but only 56% under hyperoxia (Fig. 8A–C).

To investigate proliferation we cultured myoblasts in the presence of the thymidine analogue, EdU. EdU was present for the entire duration of tissue culture, thereby cumulatively marking any nuclei undergoing or having undergone cell division, thus serving as an assay of the total number of daughter cells being generated throughout the time in culture. It is a product of both the proportion of cells that divide and of the rate of cell division. Comparing 20% oxygen with 5% oxygen, fewer EdU positive nuclei were observed in hyperoxic conditions at days 1, 2, and 3 following myofibre isolation, but differences were minimal at days 1 and 2, becoming pronounced and statistically significant only at day 3 (Fig. 8D–F). Numbers of marked nuclei increased dramatically from day 1 to day 2 and were similar at both oxygen levels, suggesting that most activated myoblasts begin proliferation within this time. The increase in number of marked nuclei from day 2 to day 3 is 2.5-fold at 20% oxygen, but 4.1-fold at 5% oxygen with an average of 11 and 29 new EdU positive nuclei per fibre being produced at 20% and 5% oxygen, respectively. This massive increase in mitotically generated nuclei over a 24-hour period cannot be explained by contribution of newly activated cells alone, so must reflect an increased rate of proliferation at physiological oxygen.

The assays of MyoD expression and of EdU incorporation suggest that hyperoxia inhibits both myoblast activation and rate of proliferation. Neither activation nor proliferation were affected by the addition of 30 nM NAC under hyperoxia (Figures 8C and 8F).

## Discussion

The relevance of *in vitro* myoblast behaviour to muscle function and disease may better be interpreted where every effort is made to keep culture conditions as close as possible to the *in vivo* environment. An understanding of the artifactual mechanistic differences in cell behaviour under physiological and super-physiological oxygen levels may improve interpretation of existing and future *in vitro* studies that neglect to mimic physiological oxygen. In the present study we investigated the hypothesis that increased mitochondrial activity is involved in the mechanism by which myoblast proliferation is inhibited under hyperoxia.

We began by confirming previous reports that primary myoblast counts in 3 day cultures of isolated myofibres are lower at 20% oxygen than at the more physiological 5% oxygen. We show that this is true both of 16–20 week mice and of older 32–40 week mice, but the aged group produced a more consistently lower cell count at 20% oxygen. Using a conditionally immortal H-2K myoblast line we demonstrated that inhibition of proliferation under hyperoxic conditions is associated with greater mitochondrial activity and, conversely, that inhibition of the mitochondrial respiratory chain or of ATP synthase activity in hyperoxic cultures each increase the rate of proliferation, this being associated with reduced levels of intracellular H_2_O_2_ and ATP. In addition, we showed that 20% oxygen represses the activation (early MyoD expression) of primary myoblasts on isolated myofibres, that it also represses proliferation rate between 2 and 3 days after isolation of the myofibre, and that none of these effects are ameliorated by administration of the oxygen scavenger n-acetyl-cysteine. Manipulation of ROS levels did not affect the rate of H-2K myoblast proliferation using NAC, the ROS spin trapping agent PBN, or H_2_O_2_ (except in association with cell death under hyperoxic conditions with high concentrations of H_2_O_2_).

The original observation that early MyoD expression is decreased in 20% oxygen was accompanied by the suggestion, made on the basis of oil red O staining, that this reflects a switch from myogenicity to adipogenesis [1], although the counts of lipid-containing cells in the original report were not numerous (0.77 and 0.27 per myofibre after one week culture at 20% and 5% oxygen respectively). Our evidence does not support this suggestion that atmospheric oxygen levels favor adipogenesis over myogenesis since we found a slightly higher fusion at 20% oxygen, and no evidence of adipogenesis. We took care to thoroughly wash myofibres in accordance with our long-established protocol and did not detect any positive oil red O staining or morphological indication of adipogenesis either in myoblast cultures after six days of proliferation or of myotubes after five days of differentiation. This accords with a recent study of cells types cultured from isolated myofibres prepared with different myofibre wash protocols, that identified adipogenic cells only when wash steps were omitted, and led the authors to suggest that previous reports of non-myogenic phenotypes observed in isolated myofibre cultures are likely the result of contamination by non-myogenic progenitors from the muscle interstitium [6]. Likewise, a study of human primary myoblasts found no evidence of oil red O staining, while expression of PPARγ, the key transcription factor regulating adipogenic differentiation, was similar at 5% and 20% oxygen [4]. We suggest that the reduced number of MyoD positive nuclei (in the present study, observed just 4 hours after myofibre isolation) reflects a decreased frequency with which quiescent satellite cells are activated. This would be consistent with the lack of effect of oxygen level on MyoD expression of proliferating human myoblasts [4] and murine C2C12 myoblasts [2], since those cells were already activated.

Our assay of cell proliferation using the thymidine analogue, EdU, shows that hyperoxia has a second, repressive effect on the rate of myoblast proliferation independent of its effect on activation. This does not manifest as reduced counts of proliferated cells until 2 to 3 days after isolation; consistent with the lag of 24 to 48 hours between exit from quiescence and the commencement of rapid proliferation [25].

Dramatically decreased fusion of C2C12 myoblasts at 20% oxygen is associated with downregulation of several myogenic transcription factors, but with no significant change in MyoD expression [2]. Our measurement of fusion index of primary myoblasts showed no evidence of such a loss but, instead, a small gain of myogenicity at 20% oxygen. A gain of myogenicity is consistent with the idea that increased mitochondrial respiratory activity pushes myoblasts [19], and stem cells in general (reviewed [26]), towards differentiation. Human primary myoblasts cultured at 20% oxygen show no detectable decrease of expression of several myogenic genes (MyHC IIa, creatine kinase, MyoD, and myogenin) [4]. This is more in keeping with the small but significant increase in fusion that we observed than with the dramatic decrease found with C2C12 cells, and raises issue of departure of the immortalized C2C12 cell line from the phenotype of the freshly isolated primary myoblast in the context of oxygen-response.

In response to hyperoxia we observed raised levels of Dioc6 signal. Since mitochondrial mass was unchanged, this reflects an increased proton gradient across the mitochondrial membrane. An inverse regulatory relationship between mitochondrial ΔΨ and proliferation rate has been described in rat L6E9 myoblasts [12], where the increase of mitochondrial ΔΨ enacted by *in vitro* manipulation of extracellular pyruvate concentration was found to induce arrest of proliferation [19]. This was associated with downregulation of proliferating cell nuclear antigen expression, increased p21 expression, and cellular hypertrophy, but not with differentiation. Thus, although high mitochondrial activity is required for myogenesis [13], [14], [15], [16], [17] and, in the present study appears to encourage myogenesis, it does not necessarily induce myogenesis in all circumstances [19].

Any of the metabolic consequences of altered mitochondrial activity may have downstream effects on the rate of cell proliferation. Those metabolites shown to influence gene expression include cytosolic Ca^2+^ [9], [10], [27], [28], NAD^+^/NADH [29], ATP [30], [31], [32], [33], [34], and ROS (reviewed [35]). In the present study, we measured two such metabolites, ATP and H_2_O_2_, a variety of ROS, finding that both are increased under hyperoxia. When oligomycin A, an inhibitor of the F0 subunit of the ATP synthase, or CCCP, a mitochondrial uncoupler, were added to the culture medium under hyperoxic conditions, we observed a decrease in levels of H_2_O_2_ and ATP, concomitant with an increased rate of cell proliferation. Recent work manipulating the redox environment of C2C12 myoblasts suggests that ROS encourage myogenesis [2]. Using NAC, we succeeded in scavenging H_2_O_2_ to the levels observed at 5% oxygen but neither H-2K nor primary myoblasts were rescued from oxygen-inhibited proliferation. Likewise, we observed no effects on proliferation rate by administration of other modifiers of ROS levels: PBN and H_2_O_2_ (except where H_2_O_2_ treatment was associated with cell toxicity). We may not have scavenged all varieties of ROS, and we did not manipulate other metabolites, and the specific downstream mechanism(s) connecting mitochondrial activity with rate of myoblast proliferation is a topic for further investigation. It is noteworthy in this respect that growth arrest induced by pyruvate-treatment of rat L6E9 myoblasts was not reversed by antioxidants, nor did oxidants induce growth arrest in control rat L6E9 myoblasts [19].

Consistent with previous findings [36], under standard tissue culture conditions (20% oxygen) we observed that numbers of myoblasts cultured from isolated murine myofibres declined with age. However, cell counts at 5% oxygen were similar at the two ages we compared. This suggests that the proliferation of myoblasts from aged mice is more readily rescued, indicating that their proliferative regulation is more sensitive to the effects of hyperoxia. This view would encourage caution in the interpretation of aging studies conducted in tissue culture. Since mitochondria accumulate DNA damage, degradation of the electron transport chain, and other defects throughout the lifespan of the animal, it suggests that the involvement of mitochondria should be explored as a clear candidate for the age-related loss of cellular vigour observed in standard tissue culture (reviewed [37]) but not *in vivo* [38]. It provides a possible explanation for the observation that primary myoblasts from old mice proliferate poorly in standard tissue culture but, when engrafted on single myofibres, contribute to muscle regeneration with the same efficiency as myoblasts from young mice [39]. It places the poor proliferation of myoblasts from old mice in standard tissue culture as an artifactual consequence of oxygen levels never experienced by these cells in their natural environment. It also raises the question of the role of changes in local oxygen tension across the phases of degeneration and regeneration in an acutely damaged region of muscle as part of a signaling system for the regulation of the stages of myogenesis. In this context, cytokine mediated control of these processes may come to play a more dominant role with increasing age, accounting for the overriding systemic influences on the efficiency of muscle repair [40].

## Supporting Information

Figure S1 **Deconvolution of CFSE data for treatment of H-2K myoblasts with Oligomycin A at 5% oxygen.** Total cell counts (plotted in black) and modeled generational subsets (coloured curves) are shown at day 0 immediately following CFSE staining and following 3 days of culture, with the concentration of Oligomycin A indicated on each relevant plot. Cell counts are also shown in tabulated form. Approximately ten thousand cells were analyzed per condition.
(TIF)

Figure S2 **Deconvolution of CFSE data for treatment of H-2K myoblasts with PBN at 20% oxygen.** Total cell counts (plotted in black) and modeled generational subsets (coloured curves) are shown at day 0 immediately following CFSE staining and following 3 days of culture, with the concentration of PBN indicated on each relevant plot. Cell counts are also shown in tabulated form. Approximately ten thousand cells were analyzed per condition.
(TIF)

Figure S3 **Deconvolution of CFSE data for treatment of H-2K myoblasts with H_2_O_2_ at 5% oxygen.** Total cell counts (plotted in black) and modeled generational subsets (coloured curves) are shown at day 0 immediately following CFSE staining and following 3 days of culture, with the concentration of H_2_O_2_ indicated on each relevant plot. Cell counts are also shown in tabulated form. Approximately ten thousand cells were analyzed per condition.
(TIF)

Figure S4 **Manipulation of ROS levels using H_2_O_2_ at 20% oxygen.** ROS levels, as indicated by the geometric mean of H_2_DCFDA fluorescence determined by FACS analysis (primary y-axis), and proliferation indices, as measured using CFSE (secondary y-axis) are plotted against concentrations H_2_O_2_ for H-2K myoblasts cultured for 3 days at 20% oxygen. Line of best fit is shown for ROS levels.
(TIF)

Figure S5 **Deconvolution of CFSE data for treatment of H-2K myoblasts with H_2_O_2_ at 20% oxygen.** Total cell counts (plotted in black) and modeled generational subsets (coloured curves) are shown at day 0 immediately following CFSE staining and following 3 days of culture, with the concentration of H_2_O_2_ indicated on each relevant plot. Cell counts are also shown in tabulated form. Approximately ten thousand cells were analyzed per condition.
(TIF)
