# Supplementary figures and images for: Atmospheric Oxygen Tension Slows Myoblast Proliferation via Mitochondrial Activation
Source: PLoS One. 2012 Aug 24;7(8):e43853. doi: 10.1371/journal.pone.0043853 (PMC3427224; doi:10.1371/journal.pone.0043853)

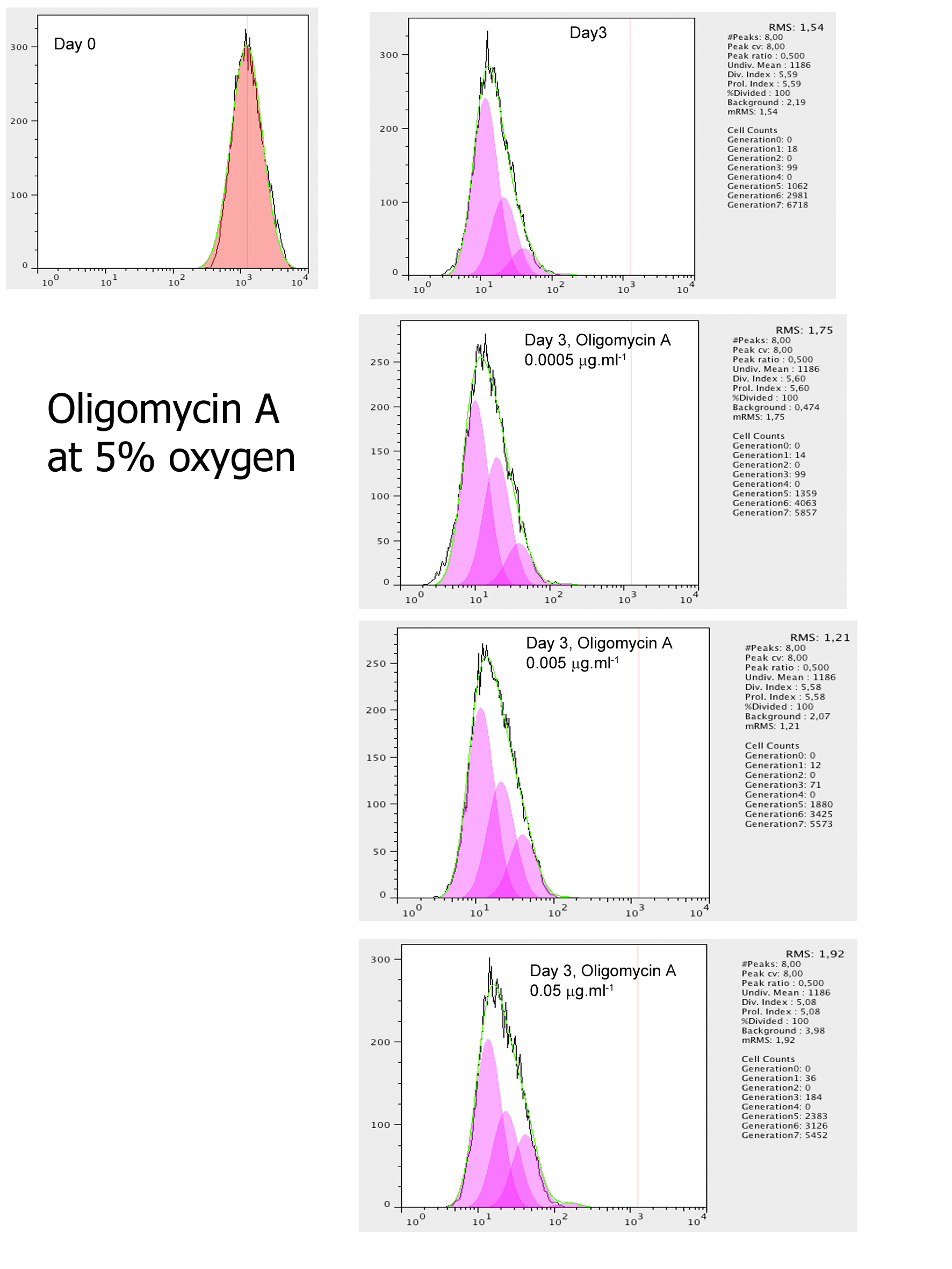

Supplement: Figure S1 — Deconvolution of CFSE data for treatment of H-2K myoblasts with Oligomycin A at 5% oxygen. Total cell counts (plotted in black) and modeled generational subsets (coloured curves) are shown at day 0 immediately following CFSE staining and following 3 days of culture, with the concentration of Oligomycin A indicated on each relevant plot. Cell counts are also shown in tabulated form. Approximately ten thousand cells were analyzed per condition. (TIF) [file pone.0043853.s001.tif]

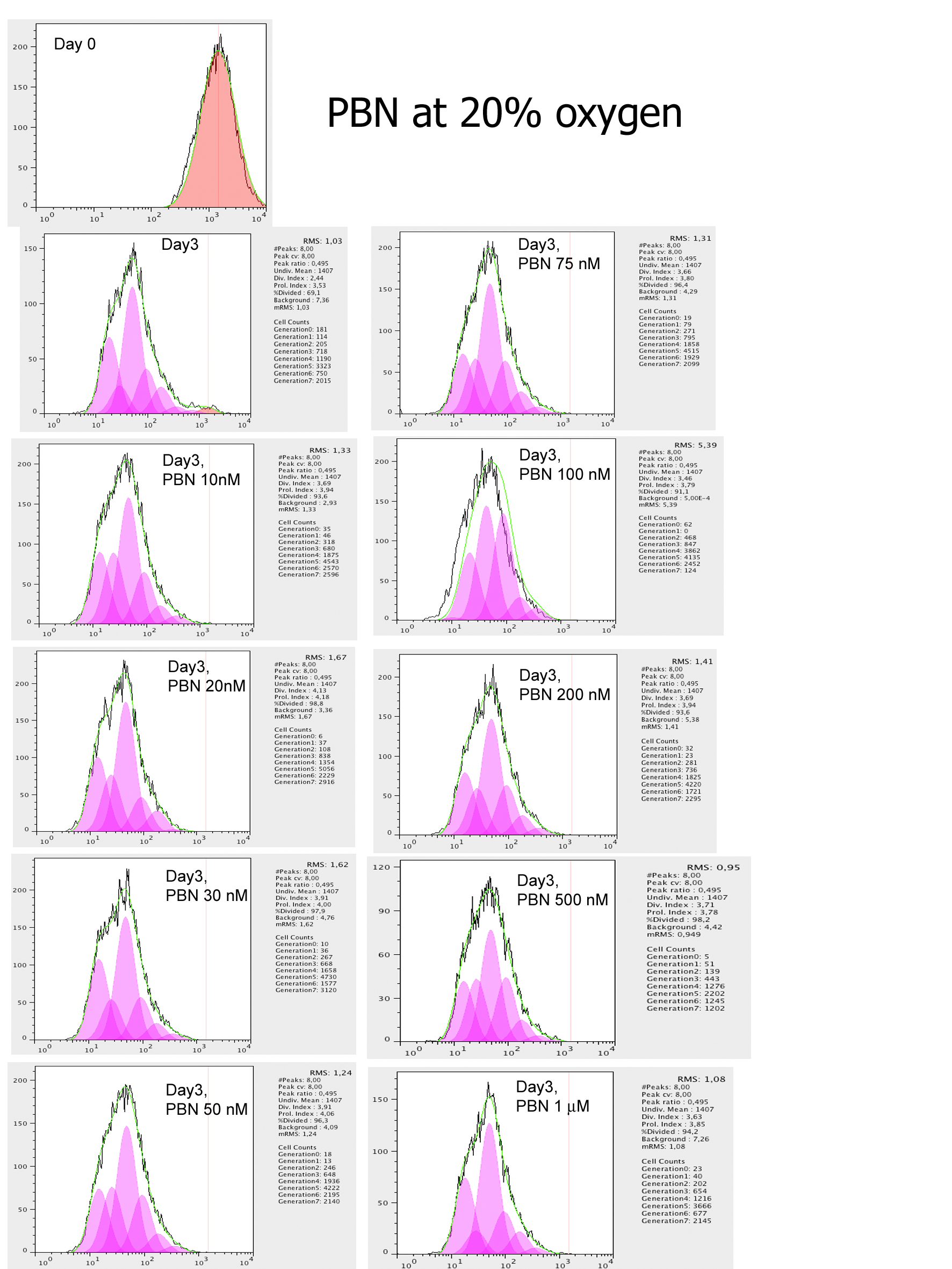

Supplement: Figure S2 — Deconvolution of CFSE data for treatment of H-2K myoblasts with PBN at 20% oxygen. Total cell counts (plotted in black) and modeled generational subsets (coloured curves) are shown at day 0 immediately following CFSE staining and following 3 days of culture, with the concentration of PBN indicated on each relevant plot. Cell counts are also shown in tabulated form. Approximately ten thousand cells were analyzed per condition. (TIF) [file pone.0043853.s002.tif]

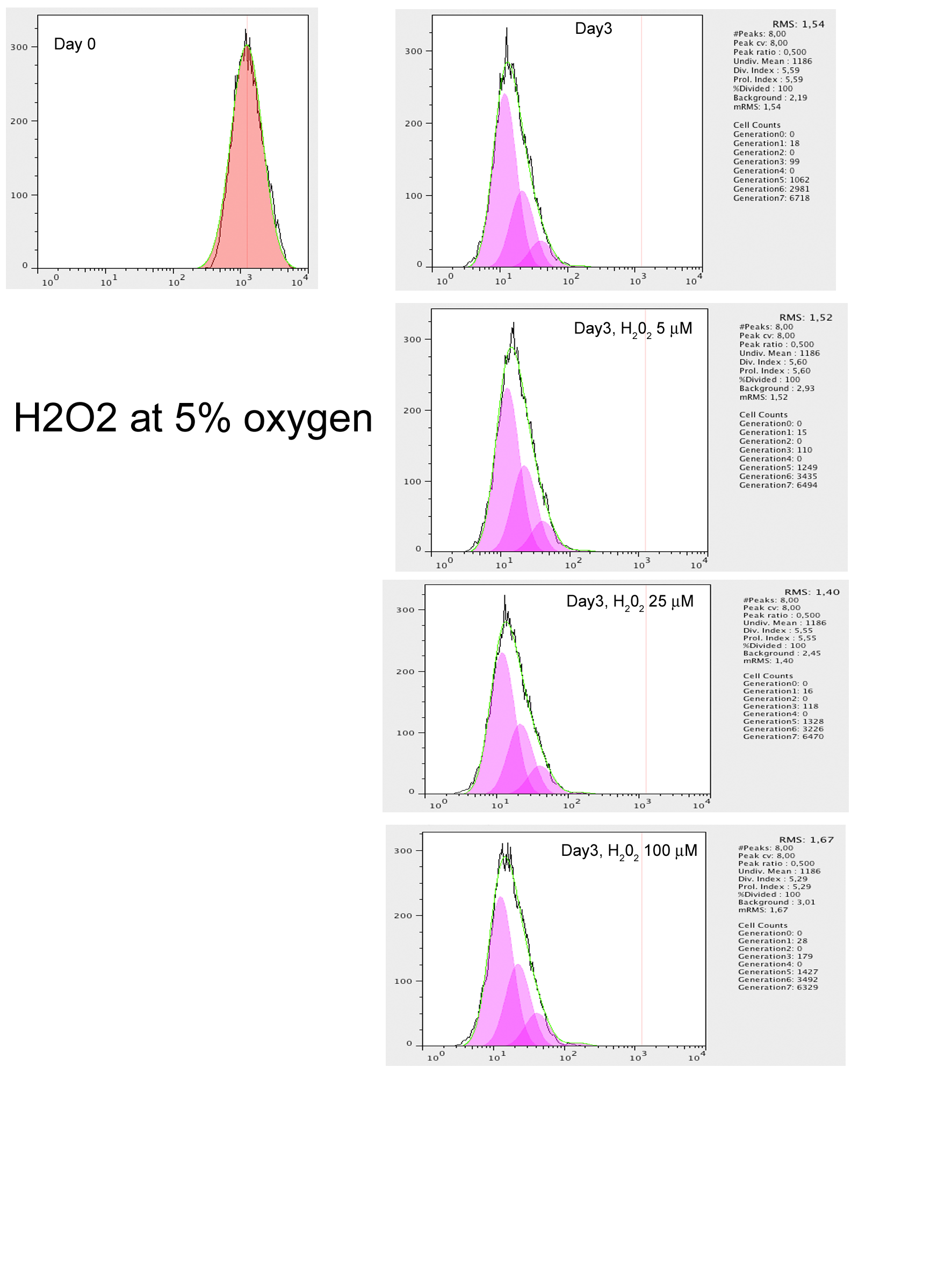

Supplement: Figure S3 — Deconvolution of CFSE data for treatment of H-2K myoblasts with H2O2 at 5% oxygen. Total cell counts (plotted in black) and modeled generational subsets (coloured curves) are shown at day 0 immediately following CFSE staining and following 3 days of culture, with the concentration of H2O2 indicated on each relevant plot. Cell counts are also shown in tabulated form. Approximately ten thousand cells were analyzed per condition. (TIF) [file pone.0043853.s003.tif]

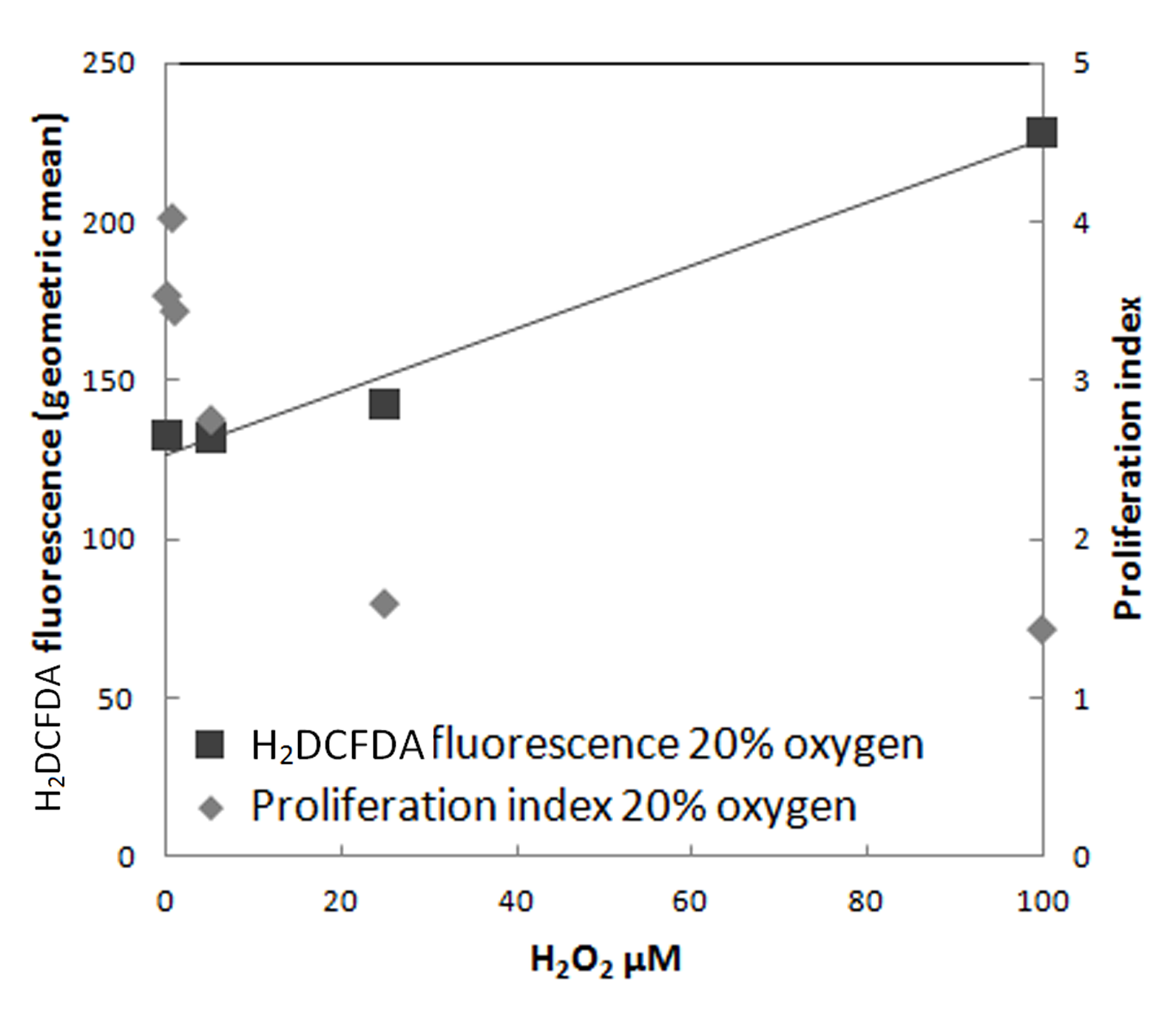

Supplement: Figure S4 — Manipulation of ROS levels using H2O2 at 20% oxygen. ROS levels, as indicated by the geometric mean of H2DCFDA fluorescence determined by FACS analysis (primary y-axis), and proliferation indices, as measured using CFSE (secondary y-axis) are plotted against concentrations H2O2 for H-2K myoblasts cultured for 3 days at 20% oxygen. Line of best fit is shown for ROS levels. (TIF) [file pone.0043853.s004.tif]

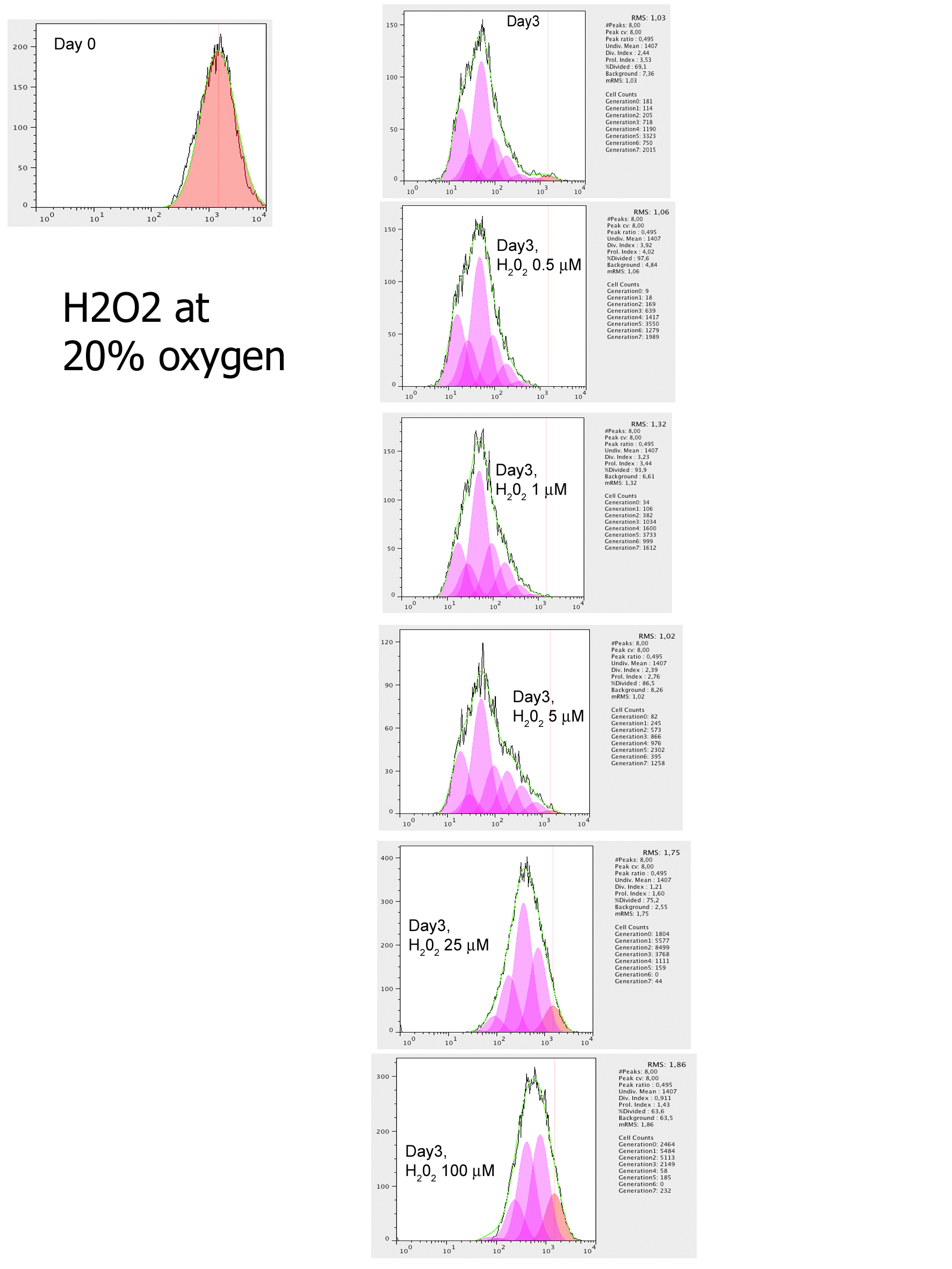

Supplement: Figure S5 — Deconvolution of CFSE data for treatment of H-2K myoblasts with H2O2 at 20% oxygen. Total cell counts (plotted in black) and modeled generational subsets (coloured curves) are shown at day 0 immediately following CFSE staining and following 3 days of culture, with the concentration of H2O2 indicated on each relevant plot. Cell counts are also shown in tabulated form. Approximately ten thousand cells were analyzed per condition. (TIF) [file pone.0043853.s005.tif]
